# Supplementary material for: Individuals with Metabolic Syndrome Show Altered Fecal Lipidomic Profiles with No Signs of Intestinal Inflammation or Increased Intestinal Permeability
Source: Metabolites. 2022 May 11;12(5):431. doi: 10.3390/metabo12050431 (PMC9143200; doi:10.3390/metabo12050431)
Supplement: Supplementary file 1 [file metabolites-12-00431-s001.zip › metabolites-1705641-supplementary.pdf]

Individuals with Metabolic Syndrome show altered fecal lipidomic profiles with no signs of intestinal inflammation or increased intestinal permeability

Coleman *et al*.,

Contents: Figure legends S1-3, Figure S1-3, Tables S1-4

Figure S1. Body Composition and Insulin resistances calculation. Graph showing (A) weight (kg); (B) body mass index (BMI); (C) Waist-to-Hip Ratio; (D) Height (cm); (E) percent of body fat; (F) Fat mass (kg); (G) Lean mass (kg); (H) HOMA-IR; and (**I**) *QUICKI*. Graphs indicate median (±minimum and maximum). Graphs indicate median (± minimum and maximum). * $p < 0.05$, ** $p < 0.01$, *** $p < 0.001$ and ns, not significant. Two-tailed unpaired Student's t tests (Figure S1 A,C,D,G,I) or two-tailed Mann–Whitney U (Figure S1 B,E,F,H).

Figure S2. Fecal lipidomics. **(A)** Plot showing the top 30 LMSD identified fecal lipids found to be the most significantly different (mean; *p*-value range: 0.008 – 0.003). SP, sphingolipids. **(B)** Three component Principal components analysis (PCA) model of hybrid metabolites. Color represents sample group, please see figure legend. **(C)** Heatmap showing unsupervised clustering analysis of samples (HC vs MetS) using the significant metabolites ($p < 0.05$) from the comparison, samples were clustered with the Complete method and Euclidian distance function.

Figure S3. Pearson's correlation coefficients between Metabolic Syndrome risk factors and fecal metabolites. Heatmap showing Pearson's r between triglyceride, HDL cholesterol, TNFα, fasting insulin, and fecal metabolites that included the

top 10 lipids identified in figure 3B, carnosine, and orotic acid. Pearson's r, 0.5 – 1 and (-0.5) – (-1) were found to be significant, $p < 0.05$ (Table S4).

Figure S1

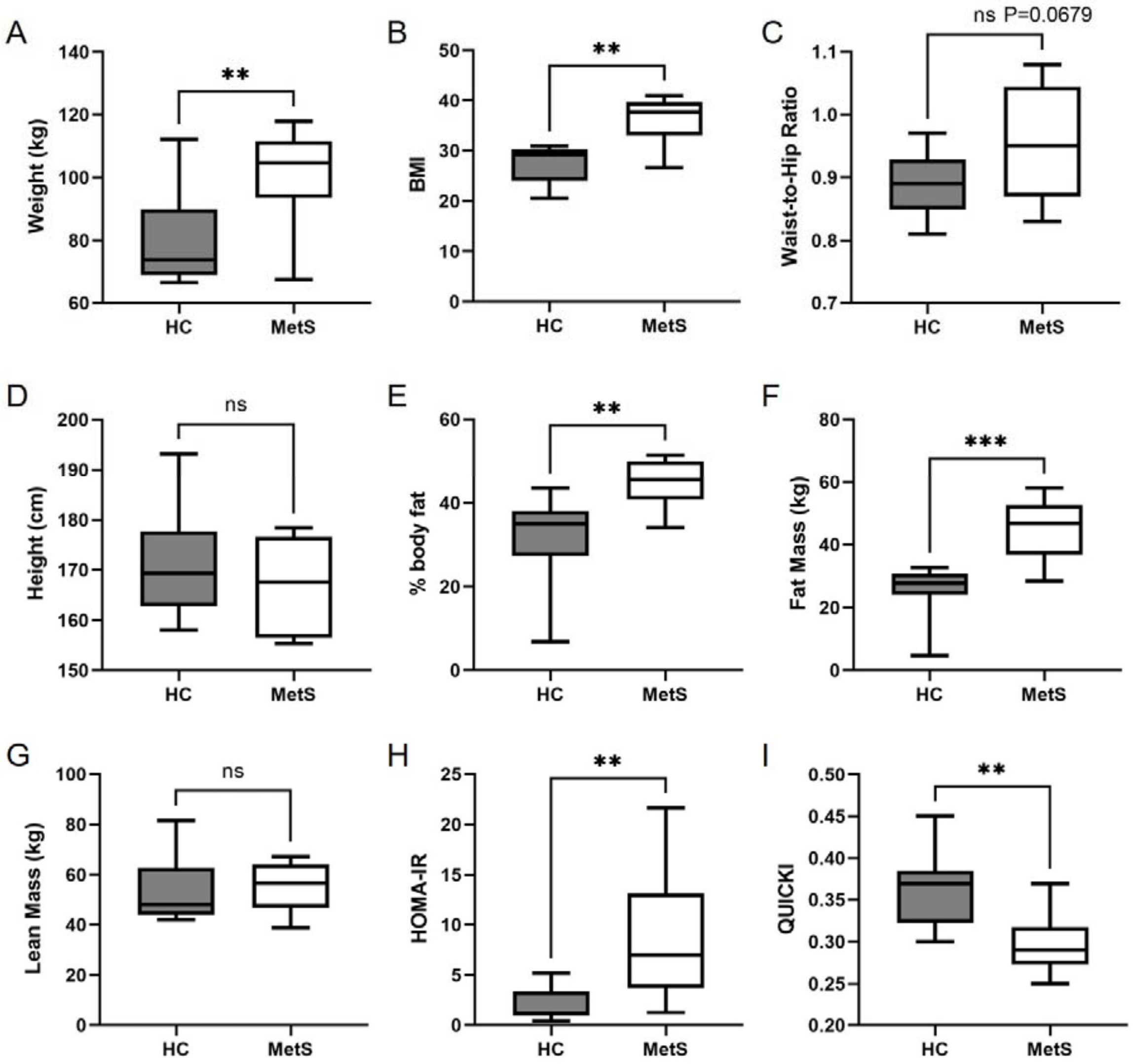

A
B
C
ns P=0.0679
Weight (kg)
BMI
Waist-to-Hip Ratio
D
E
F
ns
Height (cm)
% body fat
Fat Mass (kg)
G
H
I
ns
Lean Mass (kg)
HOMA-IR
QUICKI
HC
MetS

Figure S2

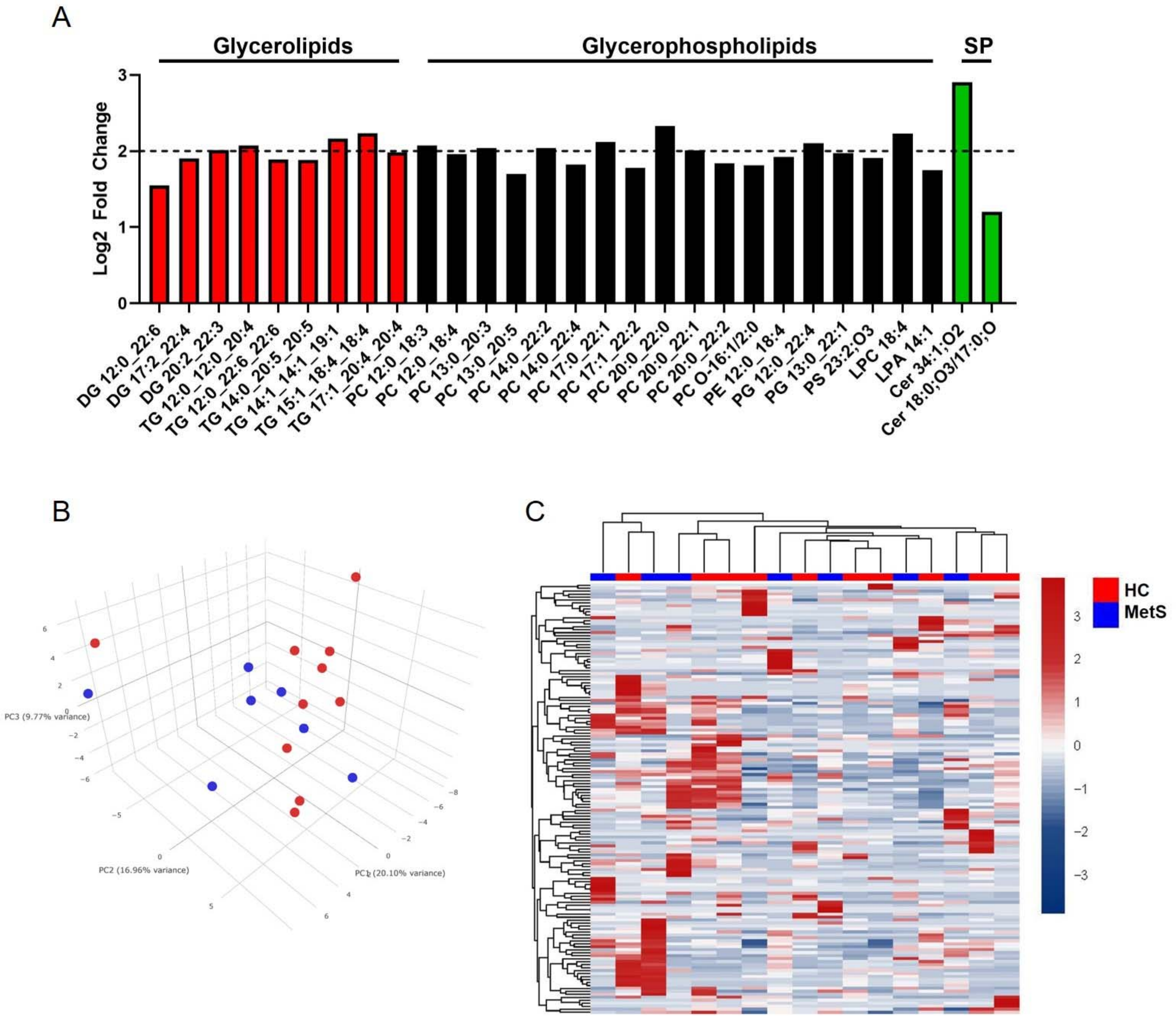
A
Glycerolipids
Glycerophospholipids
SP
Log2 Fold Change
0
1
2
3
DG 12:0_22:6
DG 17:2_22:4
DG 20:2_22:3
TG 12:0_12:0_20:4
TG 12:0_22:6_22:6
TG 14:0_20:5_20:5
TG 14:1_14:1_19:1
TG 15:1_18:4_18:4
TG 17:1_20:4_20:4
PC 12:0_18:3
PC 12:0_18:4
PC 13:0_20:3
PC 13:0_20:5
PC 14:0_22:2
PC 14:0_22:4
PC 17:0_22:1
PC 17:1_22:2
PC 20:0_22:0
PC 20:0_22:1
PC 20:0_22:2
PC O-16:1/2:0
PE 12:0_18:4
PG 12:0_22:4
PG 13:0_22:1
PS 23:2;O3
LPC 18:4
LPA 14:1
Cer 34:1;O2
Cer 18:0;O3/17:0;O
B
PC3 (9.77% variance)
PC2 (16.96% variance)
PC1 (20.10% variance)
C
HC
MetS

Figure S3

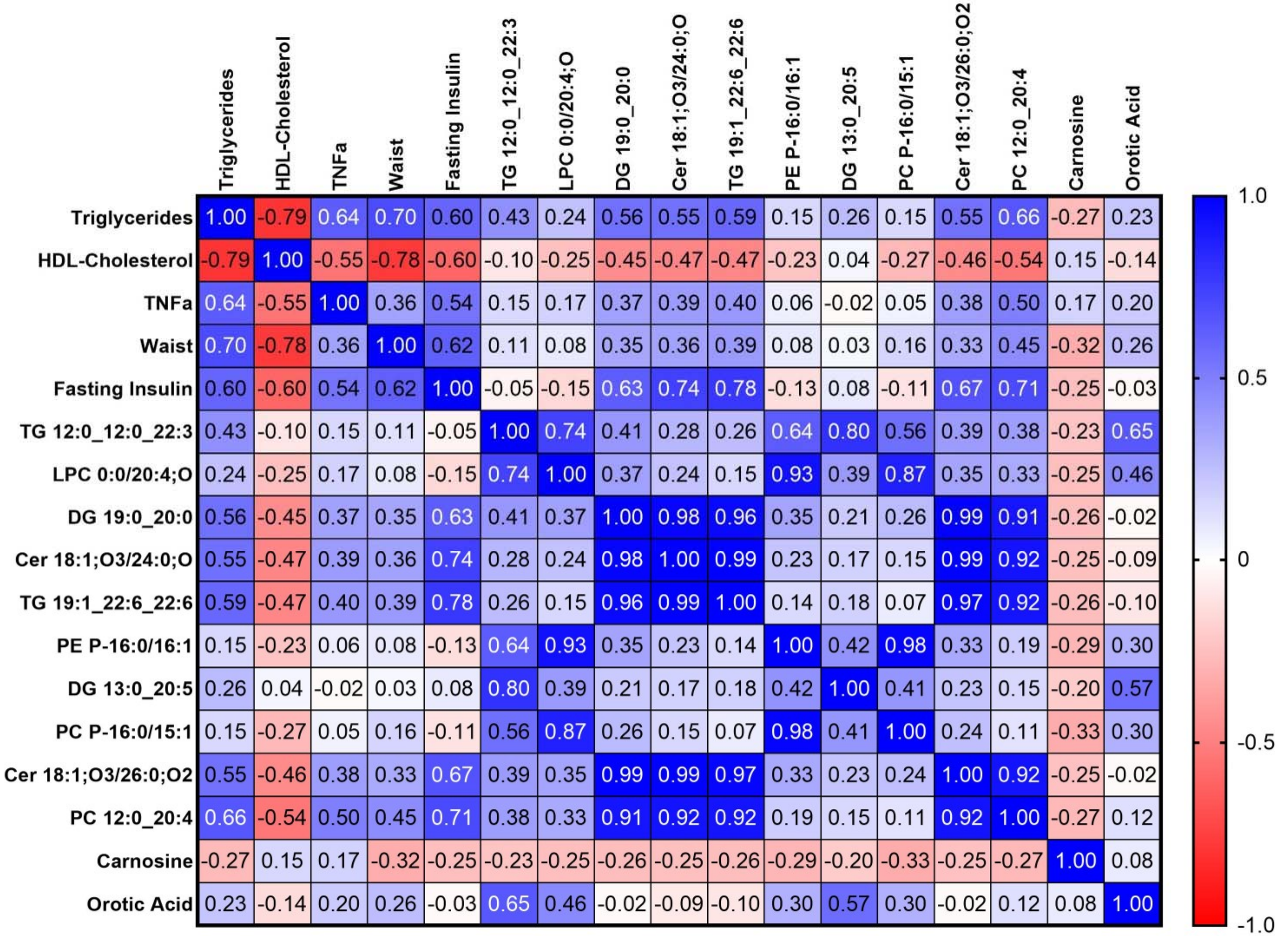
Triglycerides
HDL-Cholesterol
TNFa
Waist
Fasting Insulin
TG 12:0_12:0_22:3
LPC 0:0/20:4;O
DG 19:0_20:0
Cer 18:1;O3/24:0;O
TG 19:1_22:6_22:6
PE P-16:0/16:1
DG 13:0_20:5
PC P-16:0/15:1
Cer 18:1;O3/26:0;O2
PC 12:0_20:4
Carnosine
Orotic Acid
Triglycerides 1.00 -0.79 0.64 0.70 0.60 0.43 0.24 0.56 0.55 0.59 0.15 0.26 0.15 0.55 0.66 -0.27 0.23
HDL-Cholesterol -0.79 1.00 -0.55 -0.78 -0.60 -0.10 -0.25 -0.45 -0.47 -0.47 -0.23 0.04 -0.27 -0.46 -0.54 0.15 -0.14
TNFa 0.64 -0.55 1.00 0.36 0.54 0.15 0.17 0.37 0.39 0.40 0.06 -0.02 0.05 0.38 0.50 0.17 0.20
Waist 0.70 -0.78 0.36 1.00 0.62 0.11 0.08 0.35 0.36 0.39 0.08 0.03 0.16 0.33 0.45 -0.32 0.26
Fasting Insulin 0.60 -0.60 0.54 0.62 1.00 -0.05 -0.15 0.63 0.74 0.78 -0.13 0.08 -0.11 0.67 0.71 -0.25 -0.03
TG 12:0_12:0_22:3 0.43 -0.10 0.15 0.11 -0.05 1.00 0.74 0.41 0.28 0.26 0.64 0.80 0.56 0.39 0.38 -0.23 0.65
LPC 0:0/20:4;O 0.24 -0.25 0.17 0.08 -0.15 0.74 1.00 0.37 0.24 0.15 0.93 0.39 0.87 0.35 0.33 -0.25 0.46
DG 19:0_20:0 0.56 -0.45 0.37 0.35 0.63 0.41 0.37 1.00 0.98 0.96 0.35 0.21 0.26 0.99 0.91 -0.26 -0.02
Cer 18:1;O3/24:0;O 0.55 -0.47 0.39 0.36 0.74 0.28 0.24 0.98 1.00 0.99 0.23 0.17 0.15 0.99 0.92 -0.25 -0.09
TG 19:1_22:6_22:6 0.59 -0.47 0.40 0.39 0.78 0.26 0.15 0.96 0.99 1.00 0.14 0.18 0.07 0.97 0.92 -0.26 -0.10
PE P-16:0/16:1 0.15 -0.23 0.06 0.08 -0.13 0.64 0.93 0.35 0.23 0.14 1.00 0.42 0.98 0.33 0.19 -0.29 0.30
DG 13:0_20:5 0.26 0.04 -0.02 0.03 0.08 0.80 0.39 0.21 0.17 0.18 0.42 1.00 0.41 0.23 0.15 -0.20 0.57
PC P-16:0/15:1 0.15 -0.27 0.05 0.16 -0.11 0.56 0.87 0.26 0.15 0.07 0.98 0.41 1.00 0.24 0.11 -0.33 0.30
Cer 18:1;O3/26:0;O2 0.55 -0.46 0.38 0.33 0.67 0.39 0.35 0.99 0.99 0.97 0.33 0.23 0.24 1.00 0.92 -0.25 -0.02
PC 12:0_20:4 0.66 -0.54 0.50 0.45 0.71 0.38 0.33 0.91 0.92 0.92 0.19 0.15 0.11 0.92 1.00 -0.27 0.12
Carnosine -0.27 0.15 0.17 -0.32 -0.25 -0.23 -0.25 -0.26 -0.25 -0.26 -0.29 -0.20 -0.33 -0.25 -0.27 1.00 0.08
Orotic Acid 0.23 -0.14 0.20 0.26 -0.03 0.65 0.46 -0.02 -0.09 -0.10 0.30 0.57 0.30 -0.02 0.12 0.08 1.00
1.0
0.5
0
-0.5
-1.0

Table S1. Lipid Panel

Lipid Panel	Controls	Metabolic Syndrome	*p-value*
*Total Cholesterol (*mg/dL*)*	188.50 (51.20)	161.50 (70.70)	0.259
*HDL Cholesterol (*mg/dL*)*	60.50 (14.0)	42.50 (15.25)	**0.008
*LDL Cholesterol (*mg/dL*)*	111.00 (50.25)	78.50 (68.50)	0.217
*Triglycerides (*mg/dL*)*	100.50 (73.25)	183.50 (66.5)	**0.001

Table shows median and interquartile range. Student's t test

Table S2. Comprehensive Metabolic Panel

Comprehensive Metabolic Panel	Controls	Metabolic Syndrome	*p-value*
Sodium (mmol/L)	139.50 (3.70)	140.50 (2.50)	0.795
Potassium (mmol/L)	4.20 (0.18)	4.35 (0.48)	0.592
Chloride (mmol/L)	108.50 (5.00)	109.00 (6.50)	0.961
CO_2 (mmol/L)	23.00 (3.50)	24.50 (5.75)	0.455
Anion Gap	8.00 (3.00)	6.00 (2.75)	0.865
Calcium (mg/dL)	9.30 (0.30)	9.65 (0.83)	0.493
BUN (mg/dL)	13.50 (3.50)	12.00 (8.75)	0.683
Creatinine (mg/dL)	0.785 (0.30)	0.870 (0.32)	0.243
Total Protein (gm/dL)	7.10 (0.78)	7.55 (0.58)	0.441
Albumin (gm/dL)	3.95 (0.33)	3.90 (0.78)	0.915
Globulin (gm/dL)	3.20 (0.75)	3.50 (0.60)	0.380
Bilirubin (mg/dL)	0.65 (0.75)	0.60 (0.475)	0.306
Alk Phos (U/L)	67.00 (30.00)	92.50 (37.75)	0.079
AST (U/L)	19.50 (14.00)	21.50 (27.75)	0.770
ALT (U/L)	34.00 (22.25)	33.50 (58.00)	0.288

Table shows median and interquartile range. Student’s t test

Table S3. Putative LMSD ID of the top 30 lipids with the lowest *p*-value.

Feature ID	Observed *m/z*	Log2 Fold Change	*p-value*	Putative ID* (Category)	Main Class
**5728**	785.4944	2.166	0.003	Glycerolipids	Triacylglycerols
**5013**	698.4442	2.075	0.003	Glycerophospholipids	Diacylglycerophosphocholines
**2341**	379.249	1.746	0.003	Glycerophospholipids	Monoacylglycerophosphates
**7313**	1370.813	2.903	0.003	Sphingolipids	Gangliosides
**6227**	870.5284	2.009	0.004	Glycerophospholipids	Diacylglycerophosphocholines
**6242**	872.5436	2.329	0.004	Glycerophospholipids	Diacylglycerophosphocholines
**5992**	828.5175	2.121	0.004	Glycerophospholipids	Diacylglycerophosphocholines
**3763**	583.4164	1.548	0.004	Glycerolipids	Diacylglycerols
**5406**	741.4679	2.077	0.004	Glycerolipids	Triacylglycerols
**5393**	740.4648	2.040	0.004	Glycerophospholipids	Diacylglycerophosphocholines
**3496**	543.3401	1.759	0.005	Fatty Acyls	N-acyl amines
**5998**	829.5205	2.336	0.005	Glycerolipids	Triacylglycerols
**5717**	784.4908	2.042	0.005	Glycerophospholipids	Diacylglycerophosphocholines
**6217**	869.516	1.884	0.005	Glycerolipids	Triacylglycerols
**4984**	696.439	1.957	0.005	Glycerophospholipids	Diacylglycerophosphocholines
**5363**	736.4338	1.696	0.006	Glycerophospholipids	Diacylglycerophosphocholines
**3319**	514.4841	2.231	0.006	Glycerophospholipids	Monoacylglycerophosphocholines
**5965**	824.4862	1.776	0.006	Glycerophospholipids	Diacylglycerophosphocholines
**5676**	780.4599	1.824	0.006	Glycerophospholipids	Diacylglycerophosphocholines
**3773**	584.4232	1.202	0.006	Sphingolipid	N-acyl-4-hydroxysphinganines
**4482**	654.4183	1.921	0.006	Glycerophospholipids	Diacylglycerophosphoethanolamines
**4454**	652.4126	1.910	0.006	Glycerophospholipids	Oxidized glycerophosphoserines

**4998**	697.4416	2.015	0.006	Glycerolipids	Diacylglycerols
**5403**	741.3887	2.106	0.006	Glycerophospholipids	Diacylglycerophosphoglycerols
**6509**	913.5423	1.983	0.006	Glycerolipids	Triacylglycerols
**4468**	653.4156	1.901	0.006	Glycerolipids	Diacylglycerols
**6368**	893.5571	1.889	0.007	Glycerolipids	Triacylglycerols
**6211**	868.5124	1.835	0.007	Glycerophospholipids	Diacylglycerophosphocholines
**5549**	761.4656	1.974	0.007	Glycerophospholipids	Diacylglycerophosphoglycerols
**3344**	520.4426	1.813	0.007	Glycerophospholipids	1-alkyl,2-acylglycerophosphocholines

* Putative ID derived from LIPID MAPS® Structure Database (LMSD) utilizing observed *m/z*.

Table S4. Pearson's coefficient correlation *p*-value corresponding to figure S3.

	TG	HDL	TNFa	Waist	Fasting Insulin	TG 12:0_12:0_22:3	LPC 0:0/20:4;O	DG 19:0_20:0	Cer 18:1;O3/24:0;O	TG 19:1_22:6_22:6	PE P-16:0/16:1	DG 13:0_20:5	PC P-16:0/15:1	Cer 18:1;O3/26:0;O2	PC 12:0_20:4	Carnosine	Orotic Acid
TG		0.001	0.005	0.001	0.011	0.083	0.350	0.020	0.020	0.012	0.560	0.304	0.577	0.021	0.004	0.293	0.383
HDL	0.001		0.022	0.001	0.010	0.702	0.335	0.071	0.054	0.054	0.367	0.892	0.285	0.064	0.026	0.567	0.598
TNFa	0.005	0.022		0.151	0.024	0.574	0.504	0.146	0.125	0.110	0.806	0.935	0.849	0.135	0.042	0.513	0.437
Waist	0.001	0.001	0.151		0.007	0.663	0.757	0.170	0.154	0.116	0.757	0.907	0.531	0.192	0.072	0.206	0.318
Fasting Insulin	0.011	0.010	0.024	0.007		0.855	0.552	0.006	0.001	0.001	0.615	0.754	0.670	0.003	0.001	0.324	0.924
TG 12:0_12:0_22:3	0.083	0.702	0.574	0.663	0.855		0.001	0.106	0.272	0.314	0.005	0.001	0.019	0.126	0.132	0.372	0.004
LPC 0:0/20:4;O	0.350	0.335	0.504	0.757	0.552	0.001		0.141	0.357	0.557	0.001	0.123	0.001	0.165	0.195	0.327	0.065
DG 19:0_20:0	0.020	0.071	0.146	0.170	0.006	0.106	0.141		0.001	0.001	0.164	0.428	0.307	0.001	0.001	0.323	0.927
Cer 18:1;O3/24:0;O	0.020	0.054	0.125	0.154	0.001	0.272	0.357	0.001		0.001	0.374	0.506	0.559	0.001	0.001	0.334	0.740
TG 19:1_22:6_22:6	0.012	0.054	0.110	0.116	0.001	0.314	0.557	0.001	0.001		0.583	0.497	0.780	0.001	0.001	0.316	0.699
PE P-16:0/16:1	0.560	0.367	0.806	0.757	0.615	0.001	0.001	0.164	0.374	0.583		0.091	0.001	0.190	0.455	0.264	0.236
DG 13:0_20:5	0.304	0.892	0.935	0.907	0.754	0.001	0.123	0.428	0.506	0.497	0.091		0.103	0.375	0.564	0.437	0.016
PC P-16:0/15:1	0.577	0.285	0.849	0.531	0.670	0.019	0.001	0.307	0.559	0.780	0.001	0.103		0.344	0.677	0.201	0.234
TG 18:3_18:3_20:0	0.021	0.064	0.135	0.192	0.001	0.126	0.165	0.001	0.001	0.001	0.190	0.375	0.344		0.001	0.341	0.930
DG 21:0_22:6	0.004	0.026	0.042	0.072	0.001	0.132	0.195	0.001	0.001	0.001	0.455	0.564	0.677	0.001		0.285	0.632
Carnosine	0.293	0.567	0.513	0.206	0.324	0.372	0.327	0.323	0.334	0.316	0.264	0.437	0.201	0.341	0.285		0.774
Orotic Acid	0.383	0.598	0.437	0.318	0.924	0.004	0.065	0.927	0.740	0.699	0.236	0.016	0.234	0.930	0.632	0.774	
